# The determinants of home and nursing home death: a systematic review and meta-analysis

**DOI:** 10.1186/s12904-016-0077-8

**Published:** 2016-01-20

**Authors:** Vania Costa, Craig C. Earle, Mary Jane Esplen, Robert Fowler, Russell Goldman, Daphna Grossman, Leslie Levin, Douglas G. Manuel, Shirlee Sharkey, Peter Tanuseputro, John J. You

**Affiliations:** Health Quality Ontario, 130 Bloor Street West, 10th floor, Toronto, M5S 1 N5 ON Canada; Ontario Institute for Cancer Research, 101 College Street, Toronto, M5G 1 L7 ON Canada; de Souza Institute, University Health Network, 700 Bay Street, 19th floor, Toronto, M5G 1Z6 ON Canada; Department of Medicine and Department of Critical Care Medicine, Sunnybrook Health Sciences Centre, University of Toronto, 2075 Bayview Avenue, Toronto, M4N 3M5 ON Canada; Mount Sinai Hospital, Tammy Latner Centre for Palliative Care, 60 Murray Street, 4th Floor, Box 13, L4-000, Toronto, M5T 3 L9 ON Canada; North York General Hospital, 4001 Leslie Street, Toronto, M2K 1E1 ON Canada; MaRS Discovery District, 101 College Street, Toronto, M5G 1 L7 ON Canada; Ottawa Hospital Research Institute, 725 Parkdale Ave, Ottawa, K1Y 4E9 ON Canada; Saint Elizabeth Health Care , 90 Allstate Parkway, Suite 300, Markham, L3R 6H3 ON Canada; Departments of Medicine, and Clinical Epidemiology & Biostatistics, McMaster University, 1280 Main Street West, Hamilton, L8S 4 K1 ON Canada

**Keywords:** Determinants of place of death, Palliative care, Preference for place of death, Determinants of home death, Determinants of nursing home death

## Abstract

**Background:**

Most Canadians die in hospital, and yet, many express a preference to die at home. Place of death is the result of the interaction among sociodemographic, illness- and healthcare-related factors. Although home death is sometimes considered a potential indicator of end-of-life/palliative care quality, some determinants of place of death are more modifiable than others. The objective of this systematic review was to evaluate the determinants of home and nursing home death in adult patients diagnosed with an advanced, life-limiting illness.

**Methods:**

A systematic literature search was performed for studies in English published from January 1, 2004 to September 24, 2013 that evaluated the determinants of home or nursing home death compared to hospital death in adult patients with an advanced, life-limiting condition. The adjusted odds ratios, relative risks, and 95 % confidence intervals of each determinant were extracted from the studies. Meta-analyses were performed if appropriate. The quality of individual studies was assessed using the Newcastle-Ottawa scale and the body of evidence was assessed according to the GRADE Working Group criteria.

**Results:**

Of the 5,900 citations identified, 26 retrospective cohort studies were eligible. The risk of bias in the studies identified was considered low. Factors associated with an increased likelihood of home versus hospital death included multidisciplinary home palliative care, preference for home death, cancer as opposed to other diagnoses, early referral to palliative care, not living alone, having a caregiver, and the caregiver’s coping skills.

**Conclusions:**

Knowledge about the determinants of place of death can be used to inform care planning between healthcare providers, patients and family members regarding the feasibility of dying in the preferred location and may help explain the incongruence between preferred and actual place of death.

Modifiable factors such as early referral to palliative care, presence of a multidisciplinary home palliative care team were identified, which may be amenable to interventions that improve the likelihood of a patient dying in the preferred location. Place of death may not be a very good indicator of the quality of end-of-life/palliative care since it is determined by multiple factors and is therefore dependent on individual circumstances.

**Electronic supplementary material:**

The online version of this article (doi:10.1186/s12904-016-0077-8) contains supplementary material, which is available to authorized users.

## Background

Most Canadians die in hospital. In 2011, 65 % of deaths in Canada occurred in acute care hospitals [1], and yet, many, 63 % according to an Ontario survey [2], express a preference to die at home.

The needs of terminally ill patients vary, consequently, certain places of death may be more appropriate for some patients than others [2]. According to a conceptual model [3], place of death results from an interplay of factors that can be grouped into 3 main domains: illness (type of disease, level of disability), individual, and environment. Individual-related factors include sociodemographic characteristics and patients’ preferences with regards to place of death [3]. Environment-related factors can be divided into health care input (home care, hospital bed availability, hospital admissions); social support (living arrangements, patient’s social support network, caregiver preferences); and macrosocial factors (historical trends) [3]. It is important to note that the preference for home death may decrease with the progression of the illness [4].

Although the location of death, and home death in particular, is sometimes considered as a potential indicator of the quality of end-of-life/palliative care [5], it is possible that some determinants of place of death are more modifiable than others and a comprehensive examination of these important factors and how modifiable they are is needed.

Two systematic reviews evaluating the determinants of home death in cancer patients have been published in the past decade [3, 6], one of them evaluated a single determinant, type of cancer [6]. Several studies examining the determinants of different places of death in patients with and without malignant diseases have been published since these reviews. Studies have shown that satisfaction with end-of-life care is improved when patients die in their preferred location [7]; an understanding of the factors that influence the location of death could better inform discussions among healthcare providers, patients and their families regarding patient preferences and the feasibility of dying in the preferred location. This knowledge could also inform policy decisions aimed at improving patients’ likelihood of dying in their preferred place of death.

Accordingly, we conducted an updated systematic review of the literature to evaluate the determinants of home and nursing home death among adult patients with advanced, life-limiting malignant or non-malignant illnesses to inform discussions about preferred place of death.

## Research methods

A literature search was performed using Ovid MEDLINE, Ovid MEDLINE In-Process and Other Non-Indexed Citations, Ovid Embase, EBSCO Cumulative Index to Nursing & Allied Health Literature (CINAHL), and EBM Reviews, for studies published from January 1, 2004, and September 24, 2013. The full search strategy is available in Additional file 1. The literature search start date reflects the end of the literature search of the 2006 systematic review on the determinants of home death [3]. Title and abstracts were screened and the full text of potentially relevant articles were obtained for further assessment. Study eligibility was assessed by a single reviewer for both the title and abstract screening and full text review, however, studies with uncertain eligibility were reviewed and discussed with a second reviewer.

The conduct and reporting of this review was performed according to the Preferred Reporting Items for Systematic Reviews and Meta-Analyses: The PRISMA Statement [8]. The PRISMA checklist is available in Additional file 2.

### Eligibility criteria

We examined studies that evaluated, a priori, the determinants of death at home or nursing homes compared to hospital. Studies published in English between January 1, 2004, and September 24, 2013 in adult patients diagnosed with an advanced, life-limiting condition not expected to improve or stabilize were considered. For observational studies, only those that used multivariable analyses to adjust for potential confounders were included. Duplicate publications and studies that did not report either the adjusted odds ratio (OR) or relative risk (RR), and 95 % confidence intervals (CI) for any of the determinants of place of death specified below were excluded. Similarly, studies in which more than one place of death was combined in the results (for instance hospital, hospices and nursing home deaths combined as institutional deaths) were excluded.

The following determinants were evaluated as specified a priori: type of disease, symptoms such as pain, hospital admissions, functional status, availability of home care, palliative care in the place of residence, patient and/or family preference for place of death, living arrangements, presence and support for informal caregivers.

### Quality assessment

The risk of bias in the cohort studies included was assessed using the Newcastle-Ottawa Scale (NOS) [9]. In the NOS, a study can receive a maximum of nine stars on items related to the selection of the patient population (4 stars), the comparability of the groups (2 stars) and the assessment of the outcomes of interest (3 stars) [9]. The quality of the body of evidence for each determinant was examined according to the Grading of Recommendations Assessment, Development and Evaluation (GRADE) Working Group criteria [10]. Factors considered in the assessment included study design, risk of bias, inconsistency, indirectness, imprecision, and publication bias [10]. The overall quality was determined to be high, moderate, low, or very low using a step-wise, structural methodology.

### Data extraction

Pre-determined forms were used to extract data on study design and characteristics, patients’ characteristics, and study results. The following study characteristics were extracted: year of publication, number of participants, country where the study was conducted, setting, study design, patient population, and determinants of home and nursing home death included in the multivariable analyses. The following patient characteristics were extracted: age, sex, type of underlying disease, preferred place of death, and actual place of death.

### Data analysis

The adjusted ORs or RRs, and 95 % CIs for each determinant of home or nursing home death compared to hospital death were extracted from the studies. Heterogeneity was measured using the I^2^ statistic. [11] Meta-analyses were performed when more than one study was available for a given determinant and in the absence of considerable heterogeneity using the inverse variance method and a random effects model. Stratified analyses were performed for variables such as type of disease, setting, or country where the study was conducted if deemed necessary to explain the heterogeneity. Meta-analyses were performed using Review Manager (RevMan, version 5.2. Copenhagen: The Nordic Cochrane Centre, The Cochrane Collaboration, 2012).

## Results

**Fig. 1:**
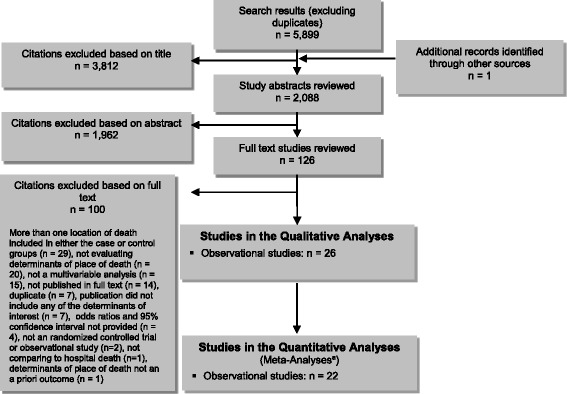
Citation Flow Chart. ^a^Meta-analyses were performed if deemed appropriate and if more than one study was available for each determinant. The number of studies included in each analysis varied according to the number of studies that reported on each determinant

The database search yielded 5,899 citations (duplicates removed), one additional study was identified through consultation with experts. Figure 1 shows the breakdown of when and for what reason citations were excluded from the analysis.

Twenty-six retrospective cohort studies using multivariable analyses met the inclusion criteria [2, 12–36]. Tables 1 and 2 show the characteristics of the patients included in these studies. Further information on study design and adjustment factors included in the multivariable analyses is available in Additional file 3.

**Table 1:** Characteristics of Patients Included in the Studies Evaluating Determinants of Home Death

Author, year, sample size, country	Patient population, age (years) male	Type of disease^a^	Place of death	NOS quality assessment scale^b^
Poulose et al. 2013 [13] *N* = 842 Singapore	• Palliative home care team recipients • ≥65: 475 (56 %) • Male: 405 (48 %)	• Cancer: 729 (87 %) • Non-cancer: 113 (13 %)	• Home: 241 (29 %) • Hospital: 452 (54 %) • Inpatient hospice: 149 (17 %)	• 7
Seow et al. 2014^c^ [36] *N* = 6,218 Canada	• Home care recipients • Median (IQR): 75: (64–84) • Male: 3,009 (48 %)	• Cancer: 4,950 (80 %) • Non-cancer: 1,268 (20 %)	• Outside of hospital: 4,828 (78 %) • Hospital: 1,390 (22 %)	• 7
Taylor et al. 2011 [17] *N* = 1,268 New Zealand	• Hospice care recipients • ≥55: 1,108 (88 %) • Male: 603 (48 %)	• Cancer: 1,036 (82 %) • Cardiovascular: 54 (4 %) • Respiratory: 45 (4 %) • Other: 120 (10 %)	• Home: 352 (28 %) • Hospital : 675 (53 %) • Nursing home: 203 (16 %)	• 7
Houttekier et al. 2011 [19] *N* = 189,884 Belgium	• General end-of-life population • ≥65: 54,311 (83 %)^d^ • Male: 32,718 (50 %)^d^	Cause of death • Cancer: 18,321 (28 %)^d^ • Cardiovascular:16,813 (26 %)^d^ • Other: 30,100 (46 %)^d^	• Home: 14,726 (23 %)^d^ • Hospital: 33,856 (52 %) ^d^ • Nursing home: 14,792 (23 %)^d^ • Other: 2,061 (3 %)^d^	• 6
Ikezaki et al. 2011 [21] *N* = 4,175 Japan	• Receiving home care from nurses • Mean: 84 ± 10^e^ • Male: 2,192 (53 %)	• Cancer: 1,664 (40 %) • Cardiovascular: 504 (12 %) • Pneumonia: 481 (12 %) • Other: 1,509 (36 %)	• Home: 1,930 (46 %) • Hospital: 2,245 (54 %)	• 7
Cardenas-Turanza et al. 2011 [22] *N* = 473 Mexico	• General end-of-life population • Mean (SD): 74 (73) • Male: 235 (50 %)	Cause of death • Cancer: 91 (19 %) • Cardiovascular: 104 (22 %) • Other: 278 (58 %)	• Home: 250 (53 %) • Hospital: 223 (47 %)	• 8
Fukui et al. 2011 [23] *N* = 568 Japan	• Receiving home palliative care from nurses • Mean (SD): 73 (12) • Male: 339 (60 %)	• Cancer: 100 %	• Home: 312 (55 %) • Hospital: 256 (45 %)	• 8
Hong et al. 2011 [12] *N* = 52,120 Singapore	• General end-of-life population • ≥65: 33,938 (65 %) • Male: 28,987 (56 %)	• Cancer: 100 %	• Home: 15,801 (30 %) • Hospital: 27,592 (53 %) • Inpatient Hospice: 5,592 (11 %) • Other: 3,135 (6 %)	• 6
Houttekier et al. 2010 [20] *N* = 1,690 Belgium	• General end-of-life population • ≥65: 1,462 (88 %) • Male: 839 (50 %)	Cause of death • Cancer: 725 (43 %) • Cardiovascular: 237 (14 %) • Other: 728 (43 %)	• Home: 402 (24 %) • Hospital: 664 (39 %) • Nursing home: 451 (27 %) • Palliative care unit: 171 (10 %)	• 7
Houttekier et al. 2010 [18] *N* = 237,579^f^ Netherlands/England	• General end-of-life population • ≥70: 131,574 (73 %) • Male: 90,619 (50 %)	Cause of death • Cancer: 170,339 (72 %) • Heart failure: 11,599 (7 %) • Other: 52,454 (22 %)	• Home: 49,036 (21 %) • Hospital: 114,401 (48 %) • Nursing home: 39,256 (17 %) • Other: 34,886 (14 %)	• 6
Tang et al. 2010 [25] *N* = 201,252 Taiwan	• General end-of-life population • ≥65: 119,690 (59 %) • Male: 129,354 (64 %)	• Cancer: 100 %	• Home: 68,139 (34 %) • Hospital: 133,113 (66 %)	• 7
Hayashi et al. 2011 [26] *N* = 99 Japan	• Home care service recipients • Mean (SD): 78 (13) • Male: 49 (50 %)	• Cancer: 38 (38 %) • Ischemic heart disease: 19 (19 %) • Other: 42 (42 %)	• Home: 40 (40 %) • Hospital: 59 (60 %)	• 5
Bell et al. 2009 [27] *N* = 1,352 United States	• General end-of-life population • Mean: 84 • Male: 100 %	Cause of death • Cancer: 337 (25 %) • Coronary: 181 (13 %) • Other: 834 (62 %)	• Home: 306 (23 %) • Hospital: 800 (59 %) • Nursing home: 246 (18 %)	• 5
Lin et al. 2007 [29] *N* = 697,814 Taiwan	• General end-of-life population • ≥75: 423,552 (61 %) • Male: 290,394 (42 %)	• Cancer: 143,529 (21 %) • Circulatory: 185,679 (27 %) • Respiratory: 85,763 (12 %) • Other: 279,126 (40 %)	• Home; 459,005 (66 %) • Hospital: 238,809 (34 %)	• 7
Gruneir et al. 2007 [30] *N* = 1,402,167 United States	• General end-of-life population • ≥75: 810,453 (58 %) • Male: 671,638 (48 %)	Cause of death • Cancer: 351,944 (25 %) • Cardiovascular: 427,661 (31 %) • Other: 623,964 (44 %)	• Home: 330,447 (24 %) • Hospital: 740,405 (53 %) • Nursing home: 331,315 (24 %)	• 7
Motiwala et al. 2006 [32] *N* = 58,689 Canada	• General end-of-life population • ≥75: 43,071 (73 %) • Male: 27,749 (47 %)	• Cancer: 19,966 (34 %) • Dementia: 16,267 (28 %) • Other: 22,302 (38 %)	Not available	• 8
Cohen et al. 2006 [33] *N* = 55,759 Belgium	• General end-of-life population • ≥65: 46,271 (83 %) • Male: 28,248 (51 %)	Cause of death • Cancer: 15,008 (27 %) • Cardiovascular: 15,846 (28 %) • Other: 27,793 (45 %)	• Home: 13,549 (24 %) • Hospital: 29,943 (54 %) • Nursing home: 11,041 (20 %) • Other: 1,115 (2 %)	• 6
Brazil et al. 2005 [2] *N* = 214 Canada	• Home palliative care recipients • ≥50 year: 100 % • Male: 142 (66 %)	• Cancer: 207 (96 %) • Non-cancer: 7 (4 %)	• Home: 120 (56 %) • Institution: 94 (44 %)	• 8
Klinkenberg et al. 2005 [34] *N* = 270 Netherlands	• General end-of-life population • ≥80: 168 (62 %) • Male: 167 (62 %)	Cause of death • Cancer: 65 (24 %) • Non-cancer: 201 (76 %)	• Home: 135 (50 %) • Hospital: 86 (32 %) • Nursing home: 46 (17 %)	• 7
Aabom et al. 2005 [35] *N* = 4,386 Denmark	• Home residents • >65: 2,979 (68 %) • Male: 2,145 (49 %)	• Cancer: 100 %	• Home: 1,221 (28 %) • Hospital: 2,412 (55 %) • Nursing home: 702 (16 %)	• 7
Fukui et al. 2004 [24] *N* = 428 Japan	• Home care recipients • Mean (SD): 75 (11) • Male: 247 (58 %)	• Cancer: 100 %	• Home: 285 (67 %) • Hospital: 143 (33 %)	• 7

*Abbreviations*: *IQR* inter-quartile range, *NOS* Newcastle-Ottawa Scale, *SD* standard deviation

^a^Cause of death was used for some of the studies (as specified), if this information was reported instead of type of disease

^b^Additional details in Additional file 4

^c^The study was originally included based on data from a 2013 conference abstract, however, the results of its subsequent publication in the peer-reviewed literature in 2014 was incorporated in our analyses. [36]

^d^2007 data shown (*N* = 65,435)

^e^non-cancer patients data shown (*N* = 2,511)

^f^Data for England and Netherlands used in our analysis as the data for Belgium may be a duplicate of another publication already included in the analysis [19]. Results for England are shown on this table

**Table 2:** Characteristics of patients included in the studies evaluating determinants of nursing home death

Author, year, sample size country	Patient population, age (years) male	Type of disease^a^	Place of death	NOS quality assessment scale^b^
Ikegami et al. 2012 [14] *N* = 1,158 Japan	• Nursing home residents • Mean (SD): 89 (8) • Male: 342 (30 %)	Cause of death • Cancer: 81 (7 %) • Cardiovascular: 220 (19 %) • Pneumonia: 237 (21 %) • Other: 620 (53 %)	• Nursing home: 548 (47 %) • Hospital: 610 (53 %)	• 6
Levy et al. 2012 [15] *N* = 7,408 United States	• Nursing home residents • Median (range): 78 (21–105) • Male: 7,224 (98 %)	Patients with different diseases, proportions not provided	• Hospital: 995 (13 %) • Nursing home: 6,413 (87 %)	• 6
Houttekier et al. 2011 [19] *N* = 79,846 Belgium	• General end-of-life population • ≥65: 54,311 (83 %)^c^ • Male: 32,718 (50 %)^c^	Cause of death • Cancer: 18,321 (28 %)^c^ • Cardiovascular:16,813 (26 %)^c^ • Other: 30,100 (46 %)^c^	• Home: 14,726 (23 %)^c^ • Hospital: 33,856 (52 %) ^c^ • Nursing home: 14,792 (23 %)^c^	• 6
Houttekier et al. 2010 [20] *N* = 1,690 Belgium	• Nursing home residents • ≥65: 1,462 (88 %) • Male: 839 (50 %)	Cause of death • Cancer: 725 (43 %) • Cardiovascular: 237 (14 %) • Other: 728 (43 %)	• Home: 402 (24 %) • Hospital: 664 (39 %) • Nursing home: 451 (27 %) • Palliative care unit: 171 (10 %)	• 7
Houttekier et al. 2010 [18] *N* = 237,579^d^ Netherlands/England	• General end-of-life population • ≥70: 131,574 (73 %) • Male: 90,619 (50 %)	Cause of death • Cancer: 170,339 (72 %) • Heart failure: 11,599 (7 %) • Other: 52,454 (22 %)	• Home: 49,036 (21 %) • Hospital: 114,401 (48 %) • Nursing home: 39,256 (17 %) • Other: 34,886 (14 %)	• 6
Bell et al. 2009 [27] *N* = 1,352 United States	• General end-of-life population • Mean: 84 • Male: 100 %	Cause of death • Cancer: 337 (25 %) • Coronary: 181 (13 %) • Other: 834 (62 %)	• Home: 306 (23 %) • Hospital: 800 (59 %) • Nursing home: 246 (18 %)	• 5
Kwak et al. 2008 [28] *N* = 30,765 United States	• Nursing home residents • Mean (SD): 86 (8) • Male: 8,306 (27 %)	Cause of death • Cancer: 1,661 (5 %) • Cardiovascular: 11,291 (37 %) • Other: 17,844 (58 %)	• Home: 615 (2 %) • Nursing home: 21,228 (69 %) • Hospital: 8,307 (27 %) • Other: 615 (2 %)	• 7
Takezako et al. 2007 [31] *N* = 86 Japan	• Nursing home residents • ≥85: 53 (62 %) • Male: 20 (23 %)	• Cancer: 3 (4 %) • Cardiovascular: 20 (23 %) • Cerebrovascular: 35 (41 %) • Other: 28 (33 %)	• Nursing home: 43 (50 %) • Hospital: 43 (50 %)	• 8
Motiwala et al. 2006 [32] *N* = 58,689 Canada	• General end-of-life population • ≥75: 43,071 (73 %) • Male: 27,749 (47 %)	• Cancer: 19,966 (34 %) • Dementia: 16,267 (28 %) • Other: 22,302 (38 %)	• Not available	• 8
Levy et al. 2004 [16] *N* = 152,494 United States	• Nursing home residents • ≥65: 146,998 (96 %) • Male: not available	• Patients with different diseases, proportions not provided	• Hospital: 51,187 (34 %) • Nursing home: 101,307 (66 %)	• 7

*Abbreviations*: *NOS* Newcastle-Ottawa Scale, *SD* standard deviation

^a^Cause of death was used for some of the studies (as specified), if this information was reported instead of type of disease

^b^Additional details in Additional file 4

^c^2007 data shown (*N* = 65,435)

^d^Data for England and Netherlands used in our analysis as the data for Belgium may be a duplicate of another publication already included in the analysis [19]. Results for England are shown on this table

The results of the studies evaluating the determinants of home and nursing home deaths are shown below.

### Determinants of home death

Twenty-one retrospective cohort studies evaluated the determinants of home versus hospital death using multivariable analyses [2, 12, 13, 17–27, 29, 30, 32–36]. The multivariable analyses were based on previously collected data from administrative databases, previous studies’ databases, caregiver surveys, or chart reviews.

The sample sizes ranged from 99 to 1,402,167 patients (patient deaths). In most studies where non-participation was reported, the rate ranged from zero to 26 % [2, 12, 13, 22–24, 29, 32, 34]. One study reported that 34 % of the patients were excluded either due to missing data or due to ineligibility, [30] and 1 study reported that 49 % of questionnaires mailed were not returned [21]. All studies identified adjusted for illness-related factors, all but one study adjusted for sociodemographic factors [26], and only 2 studies (10 %) did not adjust for healthcare service availability factors [12, 27]. Additionally, 4 (19 %) studies included patient and/or family preference for place of death in the multivariable model [2, 20, 21, 23]. Eleven (48 %) studies reported on the study time-frame – these studies used data collected during the last year of the patient’s life [2, 17, 20, 22, 23, 25, 32, 34–36]. The risk of bias in the studies was considered low, 4 (19 %) studies received 8 out of 9 stars in the NOS scale, 11 (52 %) received 7 stars, and 6 (29 %) studies were awarded 5–6 stars (Table 1, details in Additional file 4). According to the NOS assessment no serious limitations were identified in the patient selection and outcome assessment, moreover, the study results were based on multivariable analyses adjusting for possible confounders.

The studies originated in various countries and/or regions: 3 in Canada [2, 32, 36]; 8 in Asia [12, 13, 21, 23–26, 29]; 6 in Europe [18–20, 33–35]; 2 in the United States [27, 30]; 1 in Mexico [22]; and 1 in New Zealand [17]. Six studies (29 %) were specific to cancer patients [12, 21, 23–25, 35] and 8 studies (38 %) were restricted to patients receiving home care [2, 13, 17, 21, 23, 24, 26, 36]. The remainder were not specific to a disease or setting. The majority of patients included in the studies were older than 65 years and approximately half were female.

Home death occurred in 21 % to 78 % of the patients (Table 1). Four studies (19 %) reported the patient and/or family preference for place of death [2, 20, 21, 23]; of those who stated a preference, 26 % to 85 % of patients preferred a home death, as did 45 % to 65 % of family members.

**Fig. 2:**
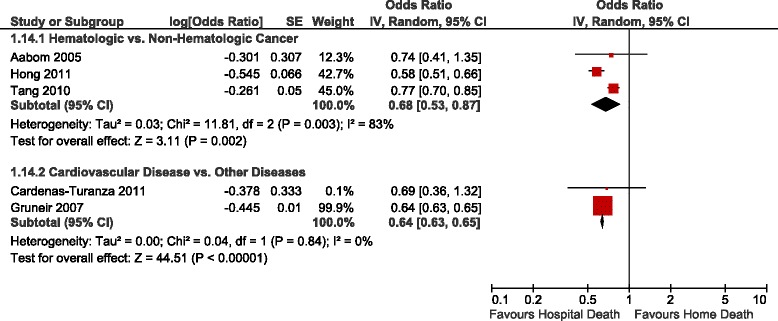
Forest Plot of the Association Between Disease Type and Home Death. Abbreviations: CI, confidence interval; df, degrees of freedom; IV, inverse variance; SE, standard error

**Fig. 3:**
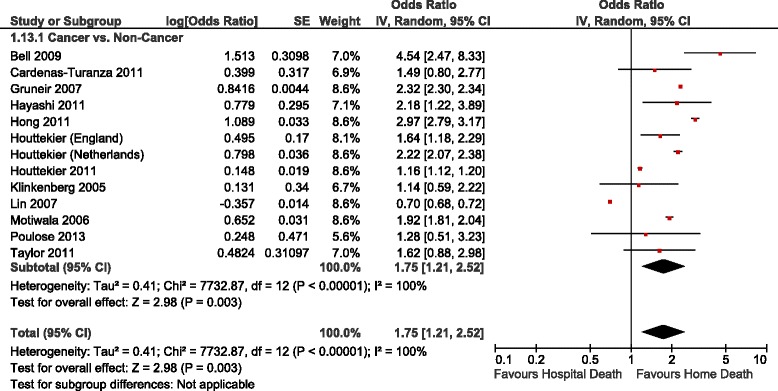
Forest Plot of the Association Between Cancer and Home Death. Abbreviations: CI, confidence interval; df, degrees of freedom; IV, inverse variance; SE, standard error

**Fig. 4:**
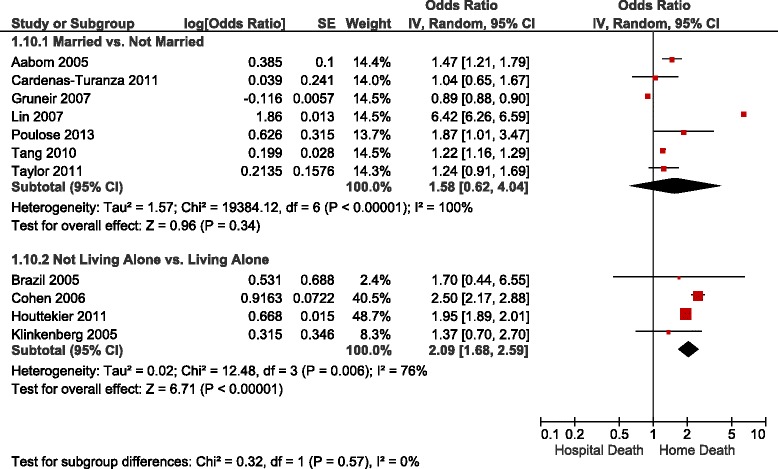
Forest Plot of the Association Between Living Arrangements and Home Death. Abbreviations: CI, confidence interval; df, degrees of freedom; IV, inverse variance; SE, standard error. Results for cancer patients used in the study by Cohen et al. [33]

Table 3 summarizes the adjusted ORs of home versus hospital death reported in the studies identified, Figs. 2, 3 and 4 show the forest plots of the meta-analyses performed. The results were stratified according to setting, i.e., whether the patients were receiving home palliative care services or not. Factors that were associated with an increased likelihood of home death included nurse and family physician home visits, multidisciplinary home palliative care, patient and family preference for home death, cancer compared to other diseases, timing of referral to palliative care, worse functional status, not living alone, presence of an informal caregiver, and caregiver coping. On the other hand, factors that decreased the likelihood of home death included hospital admissions in the last year of life, admission to a hospital with palliative care services, and some diseases such as cardiovascular versus other diseases and hematological cancers compared to solid tumours. None of the studies identified provided data on the association between symptoms and home death.

**Table 3:** Study results – determinants of home versus hospital death

Determinant	Number of Studies	Adjusted OR (95 % CI)^b^
Nurse Home Visits		
Nurse home visits in a general end-of-life population (vs. no visits)	1 study	2.78 (2.08–3.85) [35]
Nurse home visits in home care recipients (vs. no visits)	1 study	3.13 (1.08–6.21) [26]
Family Physician Home Visits		
Family physician home visits in a general end-of-life population (vs. no visits)	1 study	12.50 (8.33–16.67) [35]
Family physician home visits in home care recipients	2 studies	1.74 (1.08–2.80) [23]
		4.42 (1.46–13.36) [2]
		Pooled: 2.01 (1.30–3.12), I^2^: 57 %
Home Care Teams		
Multidisciplinary palliative home care team	2 studies	
Vs. usual care^a^		RR 2.17 (1.92–2.50) [36]
Vs. no multidisciplinary home care team		8.40 (4.70–15.10) [20]
In-Hospital Palliative Care		
In-hospital palliative support team or hospice unit (yes vs. no)	2 studies	0.34 (0.10–0.90) [20]
		0.62 (0.40–0.96) [25]
		Pooled: 0.54 (0.33–0.89), I^2^: 18 %
Preference for Home Death		
Patient preference for home death in a general end-of-life population (vs. no patient preference for home death)	1 study	14.20 (9.50–21.40) [20]
Patient preference for home death in home care recipients (vs. no patient preference for home death)	2 studies	2.04 (1.48–2.80) [21]
		2.92 (1.25–6.84) [2]
		Pooled: 2.13 (1.58–2.87), I^2^: 0
Family preference for home death vs. no family preference for home death	1 study	Non-cancer patients: 11.51 (8.56–15.99) [21]
		Cancer patients: 20.07 (12.24–32.91) [21]
Congruence between patient and family preference vs. no preference congruence	1 study	Non-cancer patients: 12.33 (9.51–15.99) [21]
		Cancer patients: 57.00 (38.79–83.76) [21]
Disease-Related		
Cancer, cardiovascular disease - See Figures 2–3		
Major acute condition vs. other conditions	1 study	0.29 (0.26–0.33) [32]
Timing of Referral to Palliative Care		
Time from referral to palliative care to death (≥1 vs. < 1 month)	1 study	2.21 (1.34–3.67) [13]
Functional Status		
Worse functional status or bedridden (vs. better functional status or not bedridden)	2 studies	2.22 (1.27–3.87) [23]
		1.82 (0.93–3.57) [34]
		Pooled: 2.05 (1.33–3.15), I^2^: 0
Prior Hospital Admission		
≥ 1 hospital admission during the last year of life (vs. no admission)	1 study	0.15 (0.07–0.30) [22]
Decision not to re-hospitalize in the event of a crisis (vs. no)	1 study	40.11 (11.81–136.26) [24]
Informal Caregiver-Related		
Presence of informal caregiver (often vs. none or sometimes)	1 study	2.30 (1.20–4.60) [20]
Low informal caregiver psychological distress during stable phase (vs. high distress)	1 study	5.41 (1.13–25.92) [24]
Informal caregiver satisfaction with support from family physician (vs. dissatisfaction)	1 study	1.62 (0.31–8.62) [2]
Informal caregiver health (excellent/very good vs. fair/poor)	1 study	0.64 (0.20–1.99) [2]
Living Arrangements		
See Figure 4		

*Abbreviations*: *CI* confidence interval, *OR* odds ratio, *RR* relative risk, *vs* versus

^a^Consisting of home care without involvement from palliative care teams [36]

^b^The reciprocal of the OR or RR and 95 % CI provided in the study was used in very few instances where necessary to ensure consistency of reporting; for instance if the OR of hospital vs. home death was provided instead of the OR of home vs. hospital death, or if the OR for non-cancer as type of disease was provided instead of cancer (OR home vs. hospital death = 1/OR hospital vs. home death) [37]

### Determinants of nursing home death

Ten observational studies using multivariable analyses evaluated the determinants of nursing home versus hospital death. [14–16, 18–20, 27, 28, 31, 32]. These were retrospective cohort studies based on previously collected data from administrative databases or chart reviews that originated in various countries and regions: 1 in Canada [32]; 3 in Europe [18–20]; 4 in the United States [15, 16, 27, 28]; and 2 in Japan [21, 31]. None of the studies were disease-specific, 6 (60 %) were restricted to nursing home residents [14–16, 20, 28, 31]; the remaining studies included a general end-of-life population some of which were nursing home residents.

The sample sizes ranged from 86 to 237,579. The non-participation rate was low in the only 2 studies that provided such data: 1 % [31] and 2 % [32]. All studies identified adjusted for illness-related factors and healthcare services availability. Eight studies (80 %) adjusted for sociodemographic factors [16, 18–20, 27, 28, 31, 32]. Additionally, 5 (50 %) studies included patient and/or family preference for place of death in the multivariable model [14–16, 20, 31]. Three (30 %) studies provided the study time-frame – these studies used data collected during the last year of the patient’s life [20, 28, 32]. The risk of bias in the studies was considered low, 2 (20 %) studies received 8 out of 9 stars in the NOS scale, 3 (30 %) received 7 stars, and 5 (50 %) studies were awarded 5–6 stars (Table 2, details in Additional file 4). According to the NOS assessment no serious limitations were identified in the patient selection and outcome assessment, moreover, the study results were based on multivariable analyses adjusting for possible confounders.

Most of the patients were older than 65 years of age and between 27 % and 100 % were male. Nursing home death occurred in 27 % to 87 % of the patients in the studies restricted to nursing home residents [14–16, 19, 28, 31] and from 17 % to 23 % in the studies in a general end-of-life population [18, 20, 27, 32]. Details in Table 2.

**Fig. 5:**
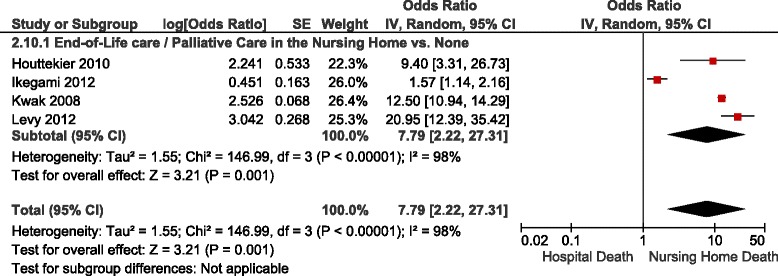
Forest Plot of the Association Between Availability of Palliative Care Services in the Nursing Home and Nursing Home vs. Hospital Death in Nursing Home Residents. Abbreviations: CI, confidence interval; df, degrees of freedom; IV, inverse variance; SE, standard error

**Fig. 6:**
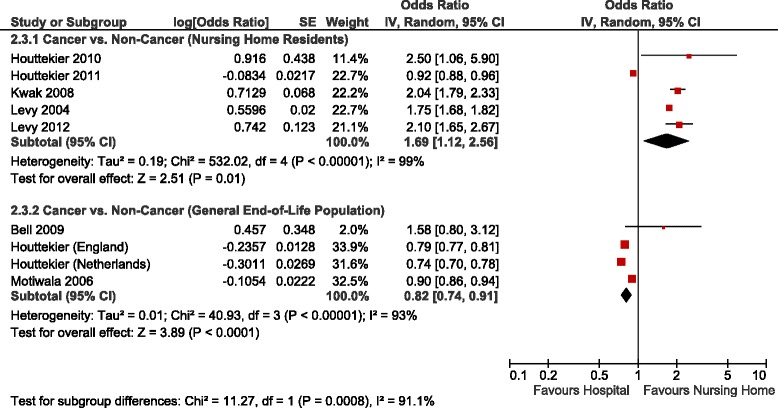
Forest Plot of the Association Between Cancer and Nursing Home vs. Hospital Death. Abbreviations: CI, confidence interval; df, degrees of freedom; IV, inverse variance; SE, standard error

**Fig. 7:**
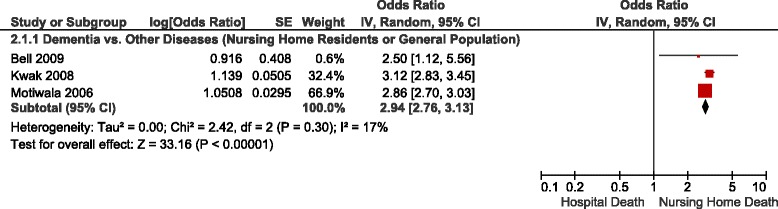
Forest Plot of the Association Between Type of Disease and Nursing Home vs. Hospital Death

Table 4 summarizes the adjusted ORs of nursing home versus hospital death reported in the studies identified, forest plots are shown in Figs. 5, 6 and 7. The results were stratified according to setting, i.e., studies restricted to nursing home residents and studies in a general end-of-life population. Factors that were associated with an increased likelihood of nursing home death included palliative care services available in the nursing home, having completed an advance directive, patient or family preference for nursing home death, some diseases such as dementia, stroke and end-stage disease, functional status, admission to a hospital-based nursing home, and a longer duration of stay at the nursing home. Among nursing home residents, with the exception of one study [19], a higher likelihood of a nursing home death was observed in patients with cancer compared to patients with other diseases across the studies identified (Figure 6) [15, 16, 20, 28]. Excluding the result of that study [19] yielded a pooled OR for nursing home versus hospital death of 1.91 (95 % CI: 1.69–2.16), and decreased the I^2^ from 99 % to 58 %. In contrast, within a general end-of-life population not restricted to nursing home residents, there was a trend towards a lower likelihood of dying in the nursing home compared to hospital in cancer versus non-cancer patients (Figure 6). Inconclusive results were observed for the association between nursing home death and living arrangements. None of the studies identified provided data on the association between symptoms and nursing home death.

**Table 4:** Study results – determinants of nursing home versus hospital death

Determinant	Number of Studies	Adjusted OR (95 % CI)^a^
End-of-Life, Palliative or Hospice Care in the Nursing Home		
See Figure 5		
Advance Directives		
Among nursing home residents	1 study	1.57 (1.35–1.82) [15]
Any advance directive (vs. no advance directive)		
Do-not-resuscitate order (yes vs. no)	1 study	3.33 (3.33–3.45) [16]
Do-not-hospitalize order (yes vs. no)	1 study	5.26 (4.76–5.88) [16]
Preference for Nursing Home Death		
Among nursing home residents		
Patient preference (yes vs. no)	1 study	10.40 (4.40–24.90) [20]
Family preference (yes vs. no)	1 study	16.62 (11.38–24.27) [14]
Disease-Related		
Cancer, dementia (vs. other diseases) See Figures 6–7		
End-stage disease (vs. non-end-stage)	1 study	3.90 (2.78–5.47) [15]
Stroke vs. other diseases (nursing home residents)	1 study	1.12 (1.06–1.18) [16]
Stroke vs. other diseases (general end-of-life population)	1 study	4.76 (2.38–9.09) [27]
Heart Failure vs. other diseases (nursing home residents)	1 study	0.75 (0.65–0.88) [15]
Diabetes vs. other diseases (nursing home residents)	2 studies	0.70 (0.61–0.81) [15]
		0.90 (0.87–0.93) [16]
		Pooled: 0.80 (0.63–1.03), I^2^: 91 %
Functional Status		
Worse functional status or bedridden vs. better functional status or not bedridden (nursing home residents)	2 studies	2.80 (0.83–9.49) [31]
		2.22 (2.04–2.38) [16]
		Pooled: 2.22 (2.07–2.38), I^2^: 0
Nursing Home Characteristics		
Hospital-based nursing home (nursing home residents)	1 study	1.21 (1.15–1.25) [16]
Full-time physician presence (nursing home residents)	1 study	3.74 (1.03–13.63 [31]
Nursing Home Stay		
1-month increment (nursing home residents)	1 study	1.01 (1.01–1.01) [15]
≥ 3 vs. < 3 months (nursing home residents)	1 study	1.44 (1.36–1.53) [28]
Living Arrangements		
Living at home before nursing home (vs. not living at home)	1 study	2.97 (0.87–10.19) [31]
Married vs. unmarried	2 studies^b^	0.35 (0.07–1.64) [31]
		1.08 (1.00–1.16) [28]

*Abbreviations*: *CI* confidence interval, *OR* odds ratio, *vs* versus

^a^The reciprocal of the OR or RR and 95 % CI provided in the study was used in very few instances where necessary to ensure consistency of reporting; for instance if the OR of hospital vs. home death was provided instead of the OR of home vs. hospital death, or if the OR for non-cancer as type of disease was provided instead of cancer (OR home vs. hospital death = 1/OR hospital vs. home death) [37]

^b^Study results were not pooled due to considerable heterogeneity, i.e., inconsistency in the direction of the study results (1 study with results favouring nursing home deaths and 1 study with results favouring hospital deaths)

### Quality assessment and grading of the evidence

The evidence identified consisted of observational studies based, in most cases, on large databases. Nevertheless, as per the NOS assessment, given that no serious limitations were identified in either the patient selection or outcome assessment, and considering that the results reported were based on multivariable analyses adjusting for factors that had previously been identified as affecting place of death, we considered the risk of bias to be low. Non-participation was not considered a serious risk of bias since, given the nature of the studies being based on large databases, non-participation was not believed to be related to either the exposure or outcome.

The quality of the body of evidence was considered low to very low. According to the GRADE Working Group criteria [10], observational studies are considered low quality. In our assessment, the quality of the evidence was downgraded to very low in a few instances due to either imprecision or inconsistencies in the study results (i.e., association between caregiver health and home death; caregiver satisfaction with support from family physician and home death; living arrangements and nursing home death). Otherwise, no downgrading of the quality of the evidence was deemed necessary as no serious limitations were identified for risk of bias, inconsistency, indirectness, or imprecision. No evidence of publication bias was observed.

## Discussion

### Summary of the evidence

In this systematic review of the literature, we identified factors that were associated with home and nursing home death. This systematic review corroborates the results of two past systematic reviews while contributing with additional results in non-cancer populations and evaluating the determinants of nursing home death. Nevertheless it should be noted that approximately a third of the studies included solely cancer patients, and 4 (14 %) other studies included at least 50 % of cancer patients. Therefore caution should be used when generalizing these results to more specific disease populations, especially as our results suggest that cancer patients may be more likely to die at home than in hospital compared to non-cancer patients.

#### Limitations

We only included studies that adjusted the results for other factors potentially associated with place of death as per a conceptual model [3]. Nonetheless, the analyses were constrained by the variables included in the original studies, for instance, although most studies adjusted for factors related to the illness, individual, and the environment, few studies adjusted for patient or family preference regarding place of death. One of the limitations inherent to this literature is the reliance on observational and often retrospective studies. The lack of randomized controlled trials (RCTs) in the literature may have been due to the fact that many determinants evaluated do not consist of interventions that patients can be assigned to. Some of the factors evaluated such as availability of palliative care in the place of residence or home visits by healthcare personnel may be amenable to an RCT design, however, possible difficulties in performing an RCT in end-of-life circumstances may have hindered this type of investigation. Considerable heterogeneity was observed in some of the meta-analyses undertaken, which may be due to the narrow confidence intervals stemming from the large sample sizes. Heterogeneity could not be further explored by meta-regression given the relatively small number of studies available for each determinant. Although studies from different countries were used in our analyses we noted that the direction of the effect for the different determinants was consistent across countries. Despite including studies from different countries, by restricting our literature search to publications in English, studies from some countries may have been unintentionally excluded, which may limit the generalizability of the results to such contexts. Our analyses did not directly focus on cultural and demographic factors such as patient age and sex, ethnicity, and socioeconomic status, however, these factors were accounted for in the analyses as most studies adjusted for at least some subset of them in their multivariable analyses.

#### Clinical practice and policy implications

Studies have shown that satisfaction with end-of-life care is improved when patients die in their preferred location [7], and information on determinants of place of death identified in these analyses may be used by policy makers to attempt to create conditions that enable patients’ dying in their preferred location.

Place of death, per se, may not be a very good indicator of the quality of end-of-life/palliative care since it is determined by a complex array of factors, some of which are not very modifiable, therefore the appropriate place of death being dependent on individual circumstances.

Some factors identified in our analyses are related to the illness and may not be modifiable, for instance type of disease, living arrangements, and the presence of symptoms that result in frequent hospital admissions. However, it may still be important to keep these factors in mind when discussing the feasibility of dying in the location of preference with patients and family members. On the other hand, if feasible, some of the factors identified can be acted upon in order to improve the likelihood that the patient’s preferences will be met. Modifiable factors include the presence of a multidisciplinary palliative care team in the home and the availability of palliative care services in the nursing home, providing support to the patient and caregiver, and a timely referral to palliative care services.

## Conclusions

The location of death depends on multiple factors that include the patients’ sociodemographic characteristics, social support, as well as illness- and healthcare-related factors. Having an understanding of these factors better informs the discussions among healthcare providers, patients and their families regarding patient preferences and the feasibility of dying in the preferred location and may perhaps help explain the incongruence between preferred and actual place of death.

Additionally, modifiable factors such as early referral to palliative care and presence of a multidisciplinary palliative care team in the patient's residence, among others were identified. Applying these interventions may improve the likelihood of a patient dying in the preferred location.

## Additional files

Additional file 1:
**Literature search strategy.** (PDF 39 kb) Additional file 2:
**PRISMA checklist.** (PDF 170 kb) Additional file 3:
**Study design and characteristics of the studies included.** (PDF 179 kb) Additional file 4:
**Newcastle-Ottawa Scale scoring.** (PDF 343 kb)

## Abbreviations

CI: Confidence interval
GRADE: Grading of Recommendations Assessment, Development and Evaluation
NOS: Newcastle-Ottawa Scale
OR: Odds ratio
PRISMA: Preferred Reporting Items for Systematic Reviews and Meta-Analyses
RCT: Randomized controlled trial
RR: Relative risk
