# Supplementary material for: The determinants of home and nursing home death: a systematic review and meta-analysis
Source: BMC Palliat Care. 2016 Jan 20;15:8. doi: 10.1186/s12904-016-0077-8 (PMC4721064; doi:10.1186/s12904-016-0077-8)
Supplement: Additional file 1: — Literature search strategy. (PDF 39 kb) [file 12904_2016_77_MOESM1_ESM.pdf]

Additional File 1: Literature Search Strategies
Search date: September 24, 2013

Database: EBM Reviews - Cochrane Database of Systematic Reviews <2005 to August 2013>, EBM Reviews - ACP Journal Club <1991 to September 2013>, EBM Reviews - Database of Abstracts of Reviews of Effects <3rd Quarter 2013>, EBM Reviews - Cochrane Central Register of Controlled Trials <August 2013>, EBM Reviews - Cochrane Methodology Register <3rd Quarter 2012>, EBM Reviews - Health Technology Assessment <3rd Quarter 2013>, EBM Reviews - NHS Economic Evaluation Database <3rd Quarter 2013>, Embase <1980 to 2013 Week 38>, Ovid MEDLINE(R) <1946 to September Week 2 2013>, Ovid MEDLINE(R) In-Process & Other Non-Indexed Citations <September 23, 2013>

Search Strategy:

#	Searches	Results
1	exp Terminal Care/	85970
2	exp Palliative Care/ use mesz,acp,cctr,coch,clcmr,dare,clhta,cleed	41033
3	exp palliative therapy/ use emez	60645
4	exp Terminally Ill/ use mesz,acp,cctr,coch,clcmr,dare,clhta,cleed	5617
5	exp terminally ill patient/ use emez	5877
6	exp terminal disease/ use emez	4477
7	exp dying/ use emez	5616
8	((End adj2 life adj2 care) or EOL care or (terminal* adj2 (care or caring or ill* or disease*)) or palliat* or dying or (Advanced adj3 (disease* or illness*)) or end stage*).ti,ab.	335051
9	or/1-8	428351
10	exp Hospices/ use mesz,acp,cctr,coch,clcmr,dare,clhta,cleed	4349
11	exp hospice/ use emez	6967
12	exp Home Care Services/ use mesz,acp,cctr,coch,clcmr,dare,clhta,cleed	41659
13	exp Home Care Agencies/ use mesz,acp,cctr,coch,clcmr,dare,clhta,cleed	1216
14	exp home care/ use emez	51971
15	exp Hospitalization/	367600
16	exp Long-Term Care/ use mesz,acp,cctr,coch,clcmr,dare,clhta,cleed	22720
17	exp Nursing Homes/ use mesz,acp,cctr,coch,clcmr,dare,clhta,cleed	32849
18	exp nursing home/ use emez	37834
19	exp Homes for the Aged/ use mesz,acp,cctr,coch,clcmr,dare,clhta,cleed	11419
20	exp home for the aged/ use emez	8622
21	((home or domicil* or communit*) adj2 (visit* or care or caring or caregiver* or health?care or assist* or aid* or agenc* or service* or rehabilitation)).ti,ab.	105277
22	(hospice* or hospital* or in?hospital or long term care facilit*).ti,ab.	1938041
23	or/10-22	2266328
24	9 and 23	70404
25	(((place or location or site) adj2 death) or ((death or dying or die) adj2 (home* or nursing home* or hospice* or hospital*))).ti,ab.	21749
26	24 or 25	88242
27	exp Health Services Accessibility/ use mesz,acp,cctr,coch,clcmr,dare,clhta,cleed	85297
28	exp Attitude to Death/	22572
29	exp Decision Making/	258108
30	exp Patient Satisfaction/	152037
31	income/	58245
32	(((determin* or factor* or indicator* or predict* or prefer*) adj2 (death or dying or die or palliative care* or terminal* ill*)) or (access* adj2 (health care or health service*))).ti,ab.	41641
33	((determin* or factor* or indicator* or predict* or prefer* or influence*) adj4 (end of life or place of death)).ti,ab.	1956

34	or/27-33	597430
35	26 and 34	10309
36	limit 35 to english language [Limit not valid in CDSR,ACP Journal Club,DARE,CCTR,CLCMR; records were retained]	9427
37	limit 36 to yr="2004 -Current" [Limit not valid in DARE; records were retained]	5681
38	remove duplicates from 37	3870

CINAHL

#	Query	Results
S1	(MH "Terminal Care+")	38,863
S2	(MH "Palliative Care")	19,643
S3	(MH "Terminally Ill Patients+")	7,655
S4	((End N2 life N2 care) or EOL care or (terminal* N2 (care or caring or ill* or disease*)) or palliat* or dying or (advanced N3 (disease* or illness*)) or end stage*)	52,080
S5	S1 OR S2 OR S3 OR S4	60,054
S6	(MH "Hospices")	2,462
S7	(MH "Home Health Care+")	32,531
S8	(MH "Home Health Agencies")	4,471
S9	(MH "Hospitalization+")	51,856
S10	(MH "Long Term Care")	18,249
S11	(MH "Nursing Homes+")	19,063
S12	((home or domicil* or communit*) N2 (visit* or care or caring or caregiver* or health care or assist* or aid* or agenc* or service* or rehabilitation))	71,862
S13	(hospice* or hospital* or in?hospital or long term care facilit*)	266,641
S14	S6 OR S7 OR S8 OR S9 OR S10 OR S11 OR S12 OR S13	387,139
S15	S5 AND S14	19,811
S16	(((place or location or site) N2 death) or ((death or dying or die) N2 (home* or nursing home* or hospice* or hospital*)))	3,092
S17	S15 OR S16	21,726
S18	(MH "Health Services Accessibility+")	47,527
S19	(MH "Attitude to Death+")	7,819
S20	(MH "Decision Making+")	62,594
S21	(MH "Patient Satisfaction")	30,524
S22	(MH "Income")	9,908
S23	(((determin* or factor* or indicator* or predict* or prefer*) N2 (death or dying or die or palliative care* or terminal* ill*)) or (access* N2 (health care or health service*)))	57,923
S24	((determin* or factor* or indicator* or predict* or prefer* or influence*) N4 (end of life or place of death))	913
S25	S18 OR S19 OR S20 OR S21 OR S22 OR S23 OR S24	162,112
S26	S17 AND S25	5,003
S27	S17 AND S25<br>Limiters - Published Date: 20040101-20131231; English Language	3,295
