# Supplementary material for: The determinants of home and nursing home death: a systematic review and meta-analysis
Source: BMC Palliat Care. 2016 Jan 20;15:8. doi: 10.1186/s12904-016-0077-8 (PMC4721064; doi:10.1186/s12904-016-0077-8)
Supplement: Additional file 3: — Study design and characteristics of the studies included. (PDF 179 kb) [file 12904_2016_77_MOESM3_ESM.pdf]

Additional File 3: Design and Characteristics of the Studies Identified

Table: Study Characteristics and Factors Adjusted for in the Multivariable Analyses

Author, Year<br>Country<br>Sample Size	Study Design<br>Data Source<br>Time Frame<br>Non-Participation	Population	Sociodemographic Factors	Illness-Related Factors	Health Services Availability	Patient or Family Preference for Place of Death
	Study Characteristics			Factors Adjusted for in the Multivariable Analyses		
Poulose et al, 2013 [1]<br>Singapore<br>N = 842	• Retrospective cohort<br>• Hospital database<br>• Time frame not specified<br>• Excluded due to missing data: 6%	• Home residents<br>• Palliative home care team recipients<br>• Referred in 2007	• Age<br>• Sex<br>• Marital status<br>• Ethnicity	• Type of disease<br>• Referral-to-death interval	• Restricted to patients receiving palliative home care	Not included
Seow, 2013 [2]<br>Canada<br>N = 6,218	• Retrospective cohort<br>• Administrative databases<br>• Time frame: last 2 weeks of life<br>• No information on non-participation	• Home residents<br>• Deaths: 2009–2011	• Age[a]<br>• Sex[a]	• Cancer[a]<br>• Comorbidities[a]	• Restricted to home care recipients<br>• Time in homecare[a]<br>• Hospital admissions[a]	Not included
Ikegami et al, 2012 [3]<br>Japan<br>N = 1,158	• Retrospective cohort<br>• Retrospective survey database<br>• Time frame not specified<br>• No information on non-participation	• Nursing home residents<br>• Deaths: 2009	Not included	• Cause of death	• Restricted to nursing home residents<br>• Physicians based in home care<br>• End-of-life care in facility	• Family preference<br>• Agreement among family members
Levy et al, 2012 [4]<br>United States<br>N = 7,408	• Retrospective cohort<br>• Administrative database<br>• Time frame not specified<br>• No information on non-participation	• Veteran Affairs nursing home residents<br>• Deaths: 2005–2007	Not included	• Type of disease<br>• End stage, not hospice<br>• Length of stay<br>• Cognitive function	• Restricted to Veteran Affairs' nursing home residents<br>• Hospice care	• Advance directives
Houttekier et al, 2011 [5]<br>Belgium<br>N = 189,884	• Retrospective cohort<br>• Administrative databases<br>• Time frame not specified<br>• No information on non-participation	• Home and nursing home residents<br>• Eligible for palliative care<br>• Deaths: 1998–2007	• Age<br>• Urbanization<br>• Living alone	• Cause of death	• Bed availability in hospital and care homes	Not included
Taylor et al, 2011 [6]<br>New Zealand<br>N = 1,268	• Retrospective cohort<br>• Chart review<br>• Time frame: last 12 months of life<br>• No information on non-participation	• Hospice care recipients<br>• Deaths: 2006–2008	• Age<br>• Sex<br>• Marital status<br>• Ethnicity	• Type of disease	• Restricted to recipients of hospice service in the community	Not included
Ikezaki et al, 2011 [7]<br>Japan<br>N = 4,175	• Retrospective cohort<br>• Data collected retrospectively from nurses<br>• Time frame: not specified<br>• Non-participation rate: 49%	• Nurse home visits recipients<br>• Deaths: 2004	• Age<br>• Sex	• Stratified by cancer/non-cancer<br>• Daily living activities	• Restricted to nurse home visit recipients<br>• Physician location<br>• Duration of home nursing service	• Patient<br>• Family<br>• Congruence patient-family

Author, Year<br>Country<br>Sample Size	Study Design<br>Data Source<br>Time Frame<br>Non-Participation	Population	Sociodemographic Factors	Illness-Related Factors	Health Services Availability	Patient or Family Preference for Place of Death
	**Study Characteristics**			**Factors Adjusted for in the Multivariable Analyses**		
Fukui et al, 2011 [8]<br>Japan<br>N = 568	• Retrospective cohort<br>• Data collected retrospectively from nurses<br>• Time frame: last 6 months of life<br>• Non-participation rate: 24%	• Nurse palliative care home visits recipients<br>• Cancer patients<br>• < 6 months expected survival<br>• Deaths: 2008	• Type of caregiver	• Restricted to cancer<br>• Functional status<br>• Unrelieved symptoms<br>• Home treatment requirement	• Restricted to home palliative care recipients<br>• Nurse visits<br>• Home team affiliated with hospital	• Patient (home care)<br>• Family (home care)
Hong et al, 2011 [9]<br>Singapore<br>N = 52,120	• Retrospective cohort<br>• Cancer registry<br>• Time frame not specified<br>• Non-participation rate: 0	• General end-of-life population<br>• Cancer patients<br>• Deaths: 2000–2009	• Age<br>• Sex<br>• Ethnicity	• Restricted to cancer<br>• Type of cancer<br>• Time between diagnosis and death<br>• Cause of death	Not included	Not included
Cardenas-Turanza et al, 2011 [10]<br>Mexico<br>N = 473	• Retrospective cohort<br>• Previous study database<br>• Time frame: < last 2 years of life<br>• Non-participation rate: 10%	• General end-of-life population<br>• Deaths: 2001–2003	• Age<br>• Marital status<br>• Education<br>• Size of city	• Cause of death<br>• Hospital stay in the last year of life	• Healthcare insurance coverage	Not included
Hayashi et al, 2011 [11]<br>Japan<br>N = 99	• Retrospective cohort<br>• Chart review<br>• Time frame not specified<br>• No information on non-participation	• Home care service recipients<br>• Deaths: 2007–2010	Not included	• Type of disease	• Restricted to home care services<br>• Home nursing visits	Not included
Houttekier et al, 2010 [12]<br>Belgium<br>N=1,690	• Retrospective cohort<br>• Previous study database<br>• Time frame: last 3 months of life<br>• No information on non-participation	• Home or nursing home residents<br>• Deaths: 2005–2006	• Age[a]<br>• Sex[a]<br>• Income<br>• Informal care during last 3 months of life<br>• Place of residence[a]	• Cause of death[a]	• Hospital bed availability<br>• Care home beds[a]<br>• Family physician involvement[a]<br>• Home care involvement[a]<br>• Palliative home care team	• Patient
Houttekier et al, 2010[b] [13]<br>N = 56,341 (Netherlands)<br>N = 181,238 (England)	• Retrospective cohort<br>• Administrative databases<br>• Time frame not specified<br>• No information on non-participation	• General end-of-life population<br>• Deaths: 2003	• Age<br>• Sex<br>• Living arrangements[a]<br>• Urban residence<br>• Income[a]	• Disease type	• Hospital bed availability<br>• Nursing home bed availability	Not included
Tang et al, 2010 [14]<br>Taiwan<br>N = 201,252	• Retrospective cohort<br>• Administrative databases<br>• Time frame: last year of life<br>• No information on non-participation	• General end-of-life population<br>• Cancer patients<br>• Deaths: 2001–2006	• Age<br>• Sex<br>• Marital status	• Restricted to cancer<br>• Type of cancer<br>• Metastasis<br>• Comorbidities<br>• Diagnosis-to-death interval	• Hospital beds<br>• Hospice care<br>• Health services in the last month of life	Not included

Author, Year<br>Country<br>Sample Size	Study Design<br>Data Source<br>Time Frame<br>Non-Participation	Population	Sociodemographic Factors	Illness-Related Factors	Health Services Availability	Patient or Family Preference for Place of Death
	Study Characteristics			Factors Adjusted for in the Multivariable Analyses		
Bell et al, 2009 [15]<br>United States<br>N = 1,352	• Retrospective cohort<br>• Previous study database<br>• Time frame not specified<br>• No information on non-participation	• General end-of-life population<br>• Japanese-American men<br>• Deaths: 1991–1999	• Age[a]<br>• Restricted to men<br>• Restricted to Japanese-American	• Cause of death<br>• Cognitive impairment[a]<br>• Functional measures[a]<br>• Last examination-to-death period[a]	Not included	Not included
Kwak et al, 2008 [16]<br>United States<br>N = 30,765	• Retrospective cohort<br>• Administrative databases<br>• Time frame: last year of life<br>• No information on non-participation	• Nursing home residents<br>• Deaths: 2000–2002	• Age<br>• Sex<br>• Marital status<br>• Ethnicity<br>• Education<br>• Urban/rural residence	• Cause of death<br>• Duration of nursing home stay	• Restricted to Medicare /Medicaid nursing home residents<br>• Hospice use	Not included
Takezako et al, 2007 [17]<br>Japan<br>N = 86	• Retrospective cohort<br>• Chart review<br>• Time frame not specified<br>• 1% excluded due to missing data	• Nursing home residents<br>• Deaths: 1999–2004	• Age<br>• Sex<br>• Marital status<br>• Living at home before nursing home	• Functional status	• Restricted to nursing home residents<br>• Full-time physician presence	• Family preference for nursing home care
Lin et al, 2007 [18]<br>Taiwan<br>N = 697,814	• Retrospective cohort<br>• Administrative databases<br>• Time frame not specified<br>• 8% excluded due to missing data	• General end-of-life population<br>• Deaths: 1995–2004	• Age<br>• Sex<br>• Marital status<br>• Urbanization<br>• Geographic location	• Type of disease<br>• Cause of death	• Bed availability	Not included
Gruneir et al, 2007 [19]<br>United States<br>N = 1,402,167	• Retrospective cohort<br>• Administrative databases<br>• Time frame not specified<br>• 34% excluded due to either missing data or lack of eligibility	• Home or nursing home residents<br>• Deaths: 1997	• Age<br>• Sex<br>• Marital status<br>• Ethnicity<br>• Education<br>• Income<br>• Urban residence	• Cause of death	• Nursing home bed availability<br>• Hospital bed availability	Not included
Motiwala et al, 2006 [20]<br>Canada<br>N = 58,689	• Retrospective cohort<br>• Administrative databases<br>• Time-frame: last year of life<br>• 2% excluded due to late claims	• General end-of-life population<br>• Deaths 2001–2002	• Age[a]<br>• Sex[a]<br>• Social deprivation<br>• Immigration status	• Disease type<br>• Comorbidities<br>• Acute care conditions	• Hospital bed availability	Not included
Cohen et al, 2006 [21]<br>Belgium<br>N = 55,759	• Retrospective cohort<br>• Administrative databases<br>• Time frame not specified<br>• No information on non-participation	• General end-of-life population<br>• Deaths: 2001	• Age<br>• Sex<br>• Education<br>• Living arrangement<br>• Urbanization	• Cause of death	• Hospital bed availability	Not included

Author, Year<br>Country<br>Sample Size	Study Design<br>Data Source<br>Time Frame<br>Non-Participation	Population	Sociodemographic Factors	Illness-Related Factors	Health Services Availability	Patient or Family Preference for Place of Death
	**Study Characteristics**			**Factors Adjusted for in the Multivariable Analyses**		
Klinkenberg et al, 2005 [22]<br>Netherlands<br>N = 270	• Retrospective cohort<br>• Previous study database<br>• Last 3 months of life<br>• Non-participation: 5%	• General end-of-life population<br>• Deaths: 1995–1999	• Age<br>• Sex<br>• Marital status<br>• Education<br>• Region	• Cause of death<br>• Cognitive decline<br>• Functional status	• Care arrangement	Not included
Brazil et al, 2005 [23]<br>Canada<br>N = 214	• Retrospective cohort<br>• Informal caregiver survey<br>• Time-frame: last month of life (some analyses)<br>• Non-participation: 33%	• Home palliative care recipient<br>• Caregivers interviewed 2000–2002	• Urban residence<br>• Not living alone<br>• Caregiver age<br>• Caregiver health	• Cancer	• Restricted to home palliative care<br>• Family physician visits<br>• Caregiver satisfaction with help from family physician	• Patient
Aabom et al, 2005 [24]<br>Danemark<br>N = 4,092	• Retrospective cohort<br>• Administrative databases<br>• Time-frame: last 3 months of life<br>• No information on non-participation	• Home residents<br>• Cancer patients<br>• Deaths: 1996–1998	• Age<br>• Sex<br>• Marital status<br>• Number of children<br>• Urban residence	• Restricted to cancer<br>• Type of cancer<br>• Time from diagnosis until death	• Restricted to those living at home<br>• Family physician home visits<br>• Contact with community nurses	Not included
Fukui et al, 2004[c] [25]<br>Japan<br>N = 428	• Home care agency survey<br>• Time frame not specified<br>• Non-participation: 26%	• Cancer patients<br>• Home care recipient > 2 weeks<br>• Deaths: 2002	• Age<br>• Caregiver's distress	• Restricted to cancer<br>• Functional status<br>• Daily infusions	• Restricted to nurse home visits<br>• Hospitalization in the event of a crisis<br>• Nursing visits	Not included
Levy et al, 2004 [26]<br>United States<br>N = 152,494	• Retrospective cohort<br>• Administrative databases<br>• Time frame not specified<br>• No information on non-participation	• Nursing home residents<br>• Admissions to nursing homes in 2001	• Age<br>• Ethnicity<br>• Rural/Urban residence	• Disease type<br>• Function<br>• Cognitive performance	• Restricted to Medicare nursing home residents<br>• Type of hospital (profit/non-profit)<br>• Size of hospital	• DNR order<br>• DNH order

Abbreviations: DNH, do-not-hospitalize; DNR, do-not-resuscitate.

[a]Variables included in the multivariate model but ORs not provided

[b]Data from Belgium not included in our analysis since it may be part of another publication already included in our analyses [5].

[c]Only the variables not included in a more recent publication [8] were extracted from this study.

References

1. Poulose JV, Do YK, Neo PSH. Association between referral-to-death interval and location of death of patients referred to a hospital-based specialist palliative care service. J Pain Symptom Manage. 2013;46(2):173-81.
2. Seow H, Brazil K, Sussman J, Pereira J, Marshall D, Austin PC, Husain A, Rangrej J, Barbera L. Impact of community based, specialist palliative care teams on hospitalisations and emergency department visits late in life and hospital deaths: a pooled analysis. BMJ. 2014;348:g3496.
3. Ikegami N, Ikezaki S. Japan's policy of promoting end-of-life care in nursing homes: impact on facility and resident characteristics associated with the site of death. Health Policy. 2012;105(2-3):303-11.
4. Levy C, Hutt E, Pointer L. Site of death among veterans living in Veterans Affairs nursing homes. J Am Med Dir Assoc. 2012;13(3):199-201.
5. Houttekier D, Cohen J, Surkyn J, Deliens L. Study of recent and future trends in place of death in Belgium using death certificate data: a shift from hospitals to care homes. BMC Public Health. 2011;11:228.
6. Taylor EJ, Ensor B, Stanley J. Place of death related to demographic factors for hospice patients in Wellington, Aotearoa New Zealand. Palliat Med. 2012;26(4):342-9.
7. Ikezaki S, Ikegami N. Predictors of dying at home for patients receiving nursing services in Japan: a retrospective study comparing cancer and non-cancer deaths. BMC Palliat Care. 2011;10(3):1-11.
8. Fukui S, Fujita J, Tsujimura M, Sumikawa Y, Hayashi Y, Fukui N. Late referrals to home palliative care service affecting death at home in advanced cancer patients in Japan: a nationwide survey. Ann Oncol. 2011;22(9):2113-20.
9. Hong CY, Chow KY, Poulose J, Jin AZ, Devi A, Chee EMF, Goh C. Place of death and its determinants for patients with cancer in Singapore: an analysis of data from the Singapore cancer registry, 2000-2009. J Palliat Med. 2011;14(10):1128-34.
10. Cardenas-Turanzas M, Torres-Vigil I, Tovalin-Ahumada H, Nates JL. Hospital versus home death: results from the Mexican health and aging study. J Pain Symptom Manage. 2011;41(5):880-92.
11. Hayashi T, Nomura H, Ina K, Kato T, Hirose T, Nonogaki Z, Suzuki Y. Place of death for the elderly in need of end-of-life home care: a study in Japan. Arch Gerontol Geriatr. 2011;53(2):242-4.
12. Houttekier D, Cohen J, Van Den Block L, Bossuyt N, Deliens L. Involvement of palliative care services strongly predicts place of death in Belgium. J Palliat Med. 2010;13(12):1461-8.
13. Houttekier D, Cohen J, Bilsen J, Addington-Hall J, Onwuteaka-Philipsen B, Deliens L. Place of death in metropolitan regions: metropolitan versus non-metropolitan variation in place of death in Belgium, the Netherlands and England. Health Place. 2010;16(1):132-9.
14. Tang ST, Huang E-W, Liu T-W, Rau K-M, Hung Y-N, Wu S-C. Propensity for home death among Taiwanese cancer decedents in 2001-2006, determined by services received at end of life. J Pain Symptom Manage. 2010;40(4):566-74.
15. Bell CL, Davis J, Harrigan RC, Somogyi-Zalud E, Tanabe MKG, Masaki KH. Factors associated with place of death for elderly Japanese-American men: the Honolulu heart program and Honolulu-Asia aging study. J Am Geriatr Soc. 2009;57(4):714-8.
16. Kwak J, Haley WE, Chiriboga DA. Racial differences in hospice use and in-hospital death among medicare and medicaid dual-eligible nursing home residents. Gerontologist. 2008;48(1):32-41.
17. Takezako Y, Tamiya N, Kajii E. The nursing home versus the hospital as the place of dying for nursing home residents in Japan. Health Policy. 2007;81(2-3):280-8.

18. Lin H-C, Lin Y-J, Liu T-C, Chen C-S, Lin C-C. Urbanization and place of death for the elderly: a 10-year population-based study. Palliat Med. 2007;21(8):705-11.
19. Gruneir A, Mor V, Weitzen S, Truchil R, Teno J, Roy J. Where people die: a multilevel approach to understanding influences on site of death in America. Med Care Res Rev. 2007;64(4):351-78.
20. Motiwala SS, Croxford R, Guerriere DN, Coyte PC. Predictors of place of death for seniors in Ontario: a population-based cohort analysis. Can J Aging. 2006;25(4):363-71.
21. Cohen J, Bilsen J, Hooft P, Deboosere P, Wal G, Deliens L. Dying at home or in an institution. Using death certificates to explore the factors associated with place of death. Health Policy. 2006;78(2-3):319-29.
22. Klinkenberg M, Visser G, Van Groenou MIB, G, Deeg DJH, Willems DL. The last 3 months of life: care, transitions and the place of death of older people. Health Soc Care Community. 2005;13(5):420-30.
23. Brazil K, Howell D, Bedard M, Krueger P, Heidebrecht C. Preferences for place of care and place of death among informal caregivers of the terminally ill. Palliat Med. 2005;19(6):492-9.
24. Aabom B, Kragstrup J, Vondeling H, Bakketeig LS, Stovring H. Population-based study of place of death of patients with cancer: implications for GPs. Br J Gen Pract. 2005;55(518):684-9.
25. Fukui S, Fukui N, Kawagoe H. Predictors of place of death for Japanese patients with advanced-stage malignant disease in home care settings: a nationwide survey. Cancer. 2004;101(2):421-9.
26. Levy CR, Fish R, Kramer AM. Site of death in the hospital versus nursing home of Medicare skilled nursing facility residents admitted under Medicare's Part A benefit. J Am Geriatr Soc. 2004;52(8):1247-54.
