# Supplementary material for: The determinants of home and nursing home death: a systematic review and meta-analysis
Source: BMC Palliat Care. 2016 Jan 20;15:8. doi: 10.1186/s12904-016-0077-8 (PMC4721064; doi:10.1186/s12904-016-0077-8)
Supplement: Additional file 4: — Newcastle-Ottawa Scale scoring. (PDF 343 kb) [file 12904_2016_77_MOESM4_ESM.pdf]

Additional file 4 – Newcastle Ottawa Quality Assessment Scale for Cohort Studies

Table: Newcastle-Ottawa Quality Assessment Scale for Cohort Studies – Scores for Included Studies

	Selection				Comparability		Outcome			
**Author, Year**	**Representativeness of the exposed cohort**	**Selection of the non-exposed cohort**	**Ascertainment of exposure**	**Demonstration that Outcome of Interest was not Present at Start of Study**	**Comparability of Cohorts on the Basis of the Design or Analysis[a]**	**Study Controls for Any Additional Factor[b]**	**Assessment of Outcome**	**Was Follow-Up Long Enough for Outcomes to Occur[c]**	**Adequacy of Follow-up of Cohorts[d]**	**Total**
Poulose et al, 2013 [1]	1	1	1	1	1	0	1	0	1	7
Seow et al, 2013 [2]	1	1	1	1	1	0	1	1	0	7
Ikegami et al, 2012 [3]	1	1	1	1	0	1	1	0	0	6
Levy et al, 2012 [4]	1	1	1	1	0	1	1	0	0	6
Taylor et al, 2011 [5]	1	1	1	1	1	0	1	1	0	7
Houttekier et al, 2011 [6]	1	1	1	1	1	0	1	0	0	6
Ikezaki et al, 2011 [7]	1	1	1	1	1	1	1	0	0	7
Cardenas-Turanza et al, 2011 [8]	1	1	1	1	1	0	1	1	1	8
Fukui et al, 2011 [9]	1	1	0	1	1	1	1	1	1	8
Hong et al, 2011 [10]	1	1	1	1	0	0	1	0	1	6
Houttekier et al, 2010 [11]	1	1	0	1	1	1	1	1	0	7
Houttekier et al, 2010 [12]	1	1	1	1	1	0	1	0	0	6

	Selection				Comparability		Outcome			
**Author, Year**	**Representativeness of the exposed cohort**	**Selection of the non-exposed cohort**	**Ascertainment of exposure**	**Demonstration that Outcome of Interest was not Present at Start of Study**	**Comparability of Cohorts on the Basis of the Design or Analysis[a]**	**Study Controls for Any Additional Factor[b]**	**Assessment of Outcome**	**Was Follow-Up Long Enough for Outcomes to Occur[c]**	**Adequacy of Follow-up of Cohorts[d]**	**Total**
Tang et al, 2010 [13]	1	1	1	1	1	0	1	1	0	7
Hayashi et al, 2011 [14]	1	1	1	1	0	0	1	0	0	5
Bell et al, 2009 [15]	1	1	1	1	0	0	1	0	0	5
Kwak et al, 2008 [16]	1	1	1	1	1	0	1	1	0	7
Lin et al, 2007 [17]	1	1	1	1	1	0	1	0	1	7
Gruneir et al, 2007 [18]	1	1	1	1	1	0	1	0	1	7
Takezako et al, 2007 [19]	1	1	1	1	1	1	1	0	1	8
Motiwala et al, 2006 [20]	1	1	1	1	1	0	1	1	1	8
Cohen et al, 2006 [21]	1	1	1	1	1	0	1	0	0	6
Brazil et al, 2005 [22]	1	1	0	1	1	1	1	1	1	8
Klinkenberg et al, 2005 [23]	1	1	0	1	1	0	1	1	1	7
Aabom et al, 2005 [24]	1	1	1	1	1	0	1	1	0	7
Fukui et al, 2004 [25]	1	1	1	1	1	0	1	0	1	7

	Selection				Comparability		Outcome			
Author, Year	Representativeness of the exposed cohort	Selection of the non-exposed cohort	Ascertainment of exposure	Demonstration that Outcome of Interest was not Present at Start of Study	Comparability of Cohorts on the Basis of the Design or Analysis[a]	Study Controls for Any Additional Factor[b]	Assessment of Outcome	Was Follow-Up Long Enough for Outcomes to Occur[c]	Adequacy of Follow-up of Cohorts[d]	Total
Levy et al, 2004 [26]	1	1	1	1	1	1	1	0	0	7

[a]A point was awarded to the study if at least 1 factor from each major domain (sociodemographic, illness-related, and health service availability) was used in the multivariable analysis.
[b]A point was awarded to the study if the multivariable analysis adjusted for patient or family preference regarding place of death.
[c]A point was awarded to the study if the follow-up was provided and was within 2 years of the patient's death.
[d]A point was awarded to the study if losses to follow-up were reported and represented less than 35% of the original study cohort.

References

1. Poulose JV, Do YK, Neo PSH. Association between referral-to-death interval and location of death of patients referred to a hospital-based specialist palliative care service. J Pain Symptom Manage. 2013;46(2):173-81.
2. Seow H, editor. Seow H. Innovative expert-consult teams: do they help keep patients at home? Hospice Palliative Care Ontario Conference; 2013.
3. Ikegami N, Ikezaki S. Japan's policy of promoting end-of-life care in nursing homes: impact on facility and resident characteristics associated with the site of death. Health Policy. 2012;105(2-3):303-11.
4. Levy C, Hutt E, Pointer L. Site of death among veterans living in Veterans Affairs nursing homes. J Am Med Dir Assoc. 2012;13(3):199-201.
5. Taylor EJ, Ensor B, Stanley J. Place of death related to demographic factors for hospice patients in Wellington, Aotearoa New Zealand. Palliat Med. 2012;26(4):342-9.
6. Houttekier D, Cohen J, Surkyn J, Deliens L. Study of recent and future trends in place of death in Belgium using death certificate data: a shift from hospitals to care homes. BMC Public Health. 2011;11:228.
7. Ikezaki S, Ikegami N. Predictors of dying at home for patients receiving nursing services in Japan: a retrospective study comparing cancer and non-cancer deaths. BMC Palliat Care. 2011;10(3):1-11.
8. Cardenas-Turanzas M, Torres-Vigil I, Tovalin-Ahumada H, Nates JL. Hospital versus home death: results from the Mexican health and aging study. J Pain Symptom Manage. 2011;41(5):880-92.
9. Fukui S, Fujita J, Tsujimura M, Sumikawa Y, Hayashi Y, Fukui N. Late referrals to home palliative care service affecting death at home in advanced cancer patients in Japan: a nationwide survey. Ann Oncol. 2011;22(9):2113-20.

10. Hong CY, Chow KY, Poulose J, Jin AZ, Devi A, Chee EMF, Goh C. Place of death and its determinants for patients with cancer in Singapore: an analysis of data from the Singapore cancer registry, 2000-2009. J Palliat Med. 2011;14(10):1128-34.
11. Houttekier D, Cohen J, Van Den Block L, Bossuyt N, Deliens L. Involvement of palliative care services strongly predicts place of death in Belgium. J Palliat Med. 2010;13(12):1461-8.
12. Houttekier D, Cohen J, Bilsen J, Addington-Hall J, Onwuteaka-Philipsen B, Deliens L. Place of death in metropolitan regions: metropolitan versus non-metropolitan variation in place of death in Belgium, the Netherlands and England. Health Place. 2010;16(1):132-9.
13. Tang ST, Huang E-W, Liu T-W, Rau K-M, Hung Y-N, Wu S-C. Propensity for home death among Taiwanese cancer decedents in 2001-2006, determined by services received at end of life. J Pain Symptom Manage. 2010;40(4):566-74.
14. Hayashi T, Nomura H, Ina K, Kato T, Hirose T, Nonogaki Z, Suzuki Y. Place of death for the elderly in need of end-of-life home care: a study in Japan. Arch Gerontol Geriatr. 2011;53(2):242-4.
15. Bell CL, Davis J, Harrigan RC, Somogyi-Zalud E, Tanabe MKG, Masaki KH. Factors associated with place of death for elderly Japanese-American men: the Honolulu heart program and Honolulu-Asia aging study. J Am Geriatr Soc. 2009;57(4):714-8.
16. Kwak J, Haley WE, Chiriboga DA. Racial differences in hospice use and in-hospital death among medicare and medicaid dual-eligible nursing home residents. Gerontologist. 2008;48(1):32-41.
17. Lin H-C, Lin Y-J, Liu T-C, Chen C-S, Lin C-C. Urbanization and place of death for the elderly: a 10-year population-based study. Palliat Med. 2007;21(8):705-11.
18. Gruneir A, Mor V, Weitzen S, Truchil R, Teno J, Roy J. Where people die: a multilevel approach to understanding influences on site of death in America. Med Care Res Rev. 2007;64(4):351-78.
19. Takezako Y, Tamiya N, Kajii E. The nursing home versus the hospital as the place of dying for nursing home residents in Japan. Health Policy. 2007;81(2-3):280-8.
20. Motiwala SS, Croxford R, Guerriere DN, Coyte PC. Predictors of place of death for seniors in Ontario: a population-based cohort analysis. Can J Aging. 2006;25(4):363-71.
21. Cohen J, Bilsen J, Hooft P, Deboosere P, Wal G, Deliens L. Dying at home or in an institution. Using death certificates to explore the factors associated with place of death. Health Policy. 2006;78(2-3):319-29.
22. Brazil K, Howell D, Bedard M, Krueger P, Heidebrecht C. Preferences for place of care and place of death among informal caregivers of the terminally ill. Palliat Med. 2005;19(6):492-9.
23. Klinkenberg M, Visser G, Van Groenou MIB, G, Deeg DJH, Willems DL. The last 3 months of life: care, transitions and the place of death of older people. Health Soc Care Community. 2005;13(5):420-30.
24. Aabom B, Kragstrup J, Vondeling H, Bakketeig LS, Stovring H. Population-based study of place of death of patients with cancer: implications for GPs. Br J Gen Pract. 2005;55(518):684-9.
25. Fukui S, Fukui N, Kawagoe H. Predictors of place of death for Japanese patients with advanced-stage malignant disease in home care settings: a nationwide survey. Cancer. 2004;101(2):421-9.
26. Levy CR, Fish R, Kramer AM. Site of death in the hospital versus nursing home of Medicare skilled nursing facility residents admitted under Medicare's Part A benefit. J Am Geriatr Soc. 2004;52(8):1247-54.
